# The acute effects of exercise on cortical excitation and psychosocial outcomes in men treated for prostate cancer: a randomized controlled trial

**DOI:** 10.3389/fnagi.2014.00332

**Published:** 2014-11-26

**Authors:** Daniel Santa Mina, Crissa L. Guglietti, Danilo R. de Jesus, Saam Azargive, Andrew G. Matthew, Shabbir M. H. Alibhai, John Trachtenberg, Jeffrey Z. Daskalakis, Paul Ritvo

**Affiliations:** ^1^Kinesiology Program, University of Guelph-HumberToronto, ON, Canada; ^2^Prostate Centre – Princess Margaret Cancer CentreToronto, ON, Canada; ^3^School of Kinesiology and Health Science, York UniversityToronto, ON, Canada; ^4^Centre for Addiction and Mental HealthToronto, ON, Canada; ^5^University of TorontoToronto, ON, Canada; ^6^University Health NetworkToronto, ON, Canada; ^7^Cancer Care OntarioToronto, ON, Canada

**Keywords:** cortical silent period, anxiety, depression, physical activity, prostate cancer, randomized controlled trial

## Abstract

**Purpose**: Regular exercise improves psychological well-being in men treated for prostate cancer (PCa). For this population and among cancer survivors in general, the effect of a single bout of exercise on self-report or objective measures of psychological well-being has not been examined. We examined the acute effect of a single bout of exercise on the cortical silent period (CSP) and on self-reported mood in men that have received treatment for PCa.

**Methods**: Thirty-six PCa survivors were randomly assigned to 60 min of low to moderate intensity exercise or to a control condition. Outcomes were assessed immediately before and after either the exercise or the control condition.

**Results**: No significant between-group differences were observed in CSP or mood were observed following the exercise session or control conditions. Participants with higher scores of trait anxiety had significantly shorter CSP at baseline, as well as those receiving androgen deprivation therapy. Age and baseline CSP had a low-moderate, but significant negative correlation. Changes in CSP following the exercise condition were strongly negatively correlated with changes in self-reported vigor.

**Conclusion**: While we did not observe any acute effect of exercise on the CSP in this population, the associations between CSP and trait anxiety, age, and vigor are novel findings requiring further examination.

**Implications for Cancer Survivors**: Exercise did not acutely affect our participants in measures of psychological well-being. Additional mechanisms to explain the chronic psychosocial benefits of exercise previously observed in men with PCa require further exploration.

**Clinicaltrials.gov Identifier:** NCT01715064 (http://clinicaltrials.gov/show/NCT01715064).

## INTRODUCTION

Prostate cancer (PCa) is the most prevalent cancer and the third leading cause of cancer-related death among Canadian men (Canadian Cancer Society’s Advisory Committee on Cancer Statistics, 2013). Fortunately, advances in detection and treatment have led to earlier diagnosis and treatment, improving 10-year survival rate to over 95 (Bill-Axelson et al., 2008). Accordingly, the population of PCa survivors is growing, but the survivorship period frequently remains fraught with treatment side effects including detriments to body composition and physical capacity, depression, anxiety, fatigue, and overall reductions in quality of life (Alibhai et al., 2006). Given that physical activity and exercise have demonstrated clinically relevant physical and psychosocial benefits for men with PCa, exercise has become vital to PCa management paradigms (Thorsen et al., 2008).

Most current research on exercise and PCa focuses on the psychosocial effects of routine exercise over several months. To date, the acute effects of exercise on psychosocial well-being in PCa patients have not been examined. In other clinical populations, single exercise bouts have improved mood, reduced depression/anxiety, and increased vigor (Hansen et al., 2001; Bartholomew et al., 2005; Peluso and Guerra de Andrade, 2005). It would be advantageous to better understand single-session effects in PCa, as programs of several months duration are composed of single bouts that may each produce important acute psychosocial benefits. Interestingly, most studies examining the exercise effects on psychosocial outcomes in PCa patients have assessed fatigue, health-related quality of life and various physical measures, whereas only a few have focused on anxiety (Carmack Taylor et al., 2006) and depression outcomes (Carmack Taylor et al., 2006; Culos-Reed et al., 2010). Furthermore, no studies have evaluated the effects of exercise on neurophysiological outcomes that may affect mood.

Our group has previously shown positive mood effects resulting from mindfulness meditation and cognitive behavioral therapy with the TMS protocol termed the CSP (Radhu et al., 2012; Guglietti et al., 2013). CSP is a non-invasive method of examining cortical inhibition of the primary motor cortex, reflecting mechanisms regulated by inhibitory GABA interneurons (del Rio and DeFelipe, 1997). The CSP is one of the most widely investigated measures of cortical inhibition, and is a quick, valid, and objective measure of how cortical physiology is implicated in psychological distress. Deficits in cortical inhibition have been observed in a range of psychiatric diagnoses such as major depressive disorder (Cryan and Kaupmann, 2005; Levinson et al., 2007), anxiety disorders (Cryan and Kaupmann, 2005), obsessive-compulsive disorder (Greenberg et al., 2000; Richter et al., 2012), schizophrenia (Daskalakis et al., 2008), and borderline personality disorder (Barnow et al., 2009).

The CSP represents the period of suppression of contralateral tonic electromyography (EMG) activity produced by cortical stimulation. Many studies report that CSP is related to the GABA_B_ receptor-mediated inhibitory activity (Werhahn et al., 1999), especially when higher stimulation intensities are used (Paulus et al., 2008). Evidence supports the hypothesis that the early part of CSP (i.e., first 50 ms) is determined by spinal mechanisms, whereas the later part is mediated by cortical inhibition (Inghilleri et al., 1993; Chen et al., 1999). CSP lengthening is indicative of potentiation of GABA_B_-mediated cortical inhibition and has been associated with reduced psychological stress (Cryan and Kaupmann, 2005), cognitive behavioral therapy (Radhu et al., 2012), mindfulness meditation (Guglietti et al., 2013), and antipsychotic medication (Liu et al., 2009). Given that the CSP is negatively correlated with mental distress and PCa patients are prone to chronic anxiety and depression, CSP may be altered in this population. Indeed, potentiation of GABA_B_ mediated inhibitory neurotransmission, measured as lengthened CSP, may be associated with symptomatic improvement and mental well-being, and may reflect benefits the PCa patient experiences regarding anxiety or depression. Lengthened CSP has already been demonstrated with high-intensity exercise and linked to improved functional performance in Parkinson’s disease; however, no psychosocial measures were recorded in this trial (Fisher et al., 2008).

Research indicates that age-related deteriorations in cortical inhibition are not necessarily pathological and may be due to normal aging. Outside of motor tasks, inhibitory control has been shown to require increased prefrontal activation measured using BOLD activity in order to compensate for the differences in older adults as compared to younger adults (Nielson et al., 2002). However, most of this research assumes that the typical markers for cortical inhibition related declines from aging are decreased reaction time and decreased motor coordination. In attention-demanding reaction time tasks that require GABA_A_ pathways, EEG recordings from older adults exhibited stimulus-preceding negativity and attenuated contingent negative variation (Hillman et al., 2002) and stymied contingent negative variation amplitudes after the warning signal but preceding the imperative signal and execution of a motor task (Sterr and Dean, 2008). Furthermore, TMS applied to M1 during the waiting period demonstrated neural compensation by older individuals in order to achieve similar reaction times when compared to their younger counterparts, presumably by an increased recruitment of GABA_A_ pathways (Fujiyama et al., 2012). Because the inhibition of M1 is predominantly controlled by prefrontal cortex/premotor regions, intracortical explanations for slowed reaction times due to aging are likely to be found in the aforementioned brain regions. This claim is further supported by Buch et al. (2010) and Neubert et al. (2010) who used diffusion-tensor imaging to show that specific structures that connect prefrontal cortex with M1 (central-prefrontal/premotor and pre-SMA, respectively) had deteriorated in proportion to the measured reductions in cortical inhibition.

Extending this theory of reduced cortical inhibition in the aging population to the field of exercise physiology can be found in the following studies on motor coordination in older adults. TMS studies on complex motor tasks of low coordination stability illustrate the recruitment of inhibitory processes (i.e., CSP) that are normally observed in younger adults but seem to be absent in older adults; participants who are able to register greater CSP measurements are the ones capable of successfully accomplishing the above mentioned motor tasks (non-isodirectional patterns with ipsilateral limbs; Siebner et al., 1998; Fujiyama et al., 2009). Motor coordination was shown to deteriorate in tasks of higher speeds, especially with regards to movement variability, phase wandering, and phase transitions. The authors suggested that this is likely modulated by GABA_B_ pathways. However, this relationship between the practice of exercise gross motor tasks and with cortical inhibition particularly in older adults who are expected to have declines in this area remains unclear. Accordingly, the objectives of this study were to (1) examine the effects of a single bout of exercise on the CSP and self-report measures of mood in men who have been treated for PCa; and (2) assess the relationship between post-exercise changes in CSP and mood.

## MATERIALS AND METHODS

### STUDY DESIGN

This study was a prospective, RCT of a single bout of exercise versus a non-exercise (control) condition in PCa patients that have received curative treatment for PCa. Concealed randomization was conducted using sequentially numbered opaque envelopes containing group assignments and was provided to participants following the baseline assessment. Training staff and participants were unblinded to group allocation; however, outcome assessors were blinded. The trial was approved by the research ethics review committees at participating institutions and all participants provided written informed consent prior to participation.

### PARTICIPANTS

Eligible patients were approached for participation by a research coordinator following a urology clinic appointment at the Princess Margaret Cancer Centre in Toronto, ON, Canada from June to October, 2010. Patients could also respond to study information posters located in the clinic waiting areas. Patients were eligible if they: (i) had histologically confirmed PCa; (ii) were ≥6 months post-curative therapy for PCa (radical prostatectomy or radiation therapy) with or without adjuvant ADT; (iii) were willing and able to provide informed consent; (iv) if metastatic disease was present, they were physically asymptomatic (e.g., no bone pain); (v) had no contraindications to exercise; (vi) were between ages 45 and 85 years; (vii) were not diagnosed with psychotic, addictive, or major cognitive disorders or had a history of chronic usage of psychotropic medication (anti-depressants, anxiolytics, anti-psychotics, benzodiazepines, etc.); (viii) had no contraindications to magnetic exposure; (ix) had no prior history of seizures or diagnosis of epilepsy; and (x) were right-hand dominant.

*A priori*, we calculated the sample size necessary to detect a significant, clinically important difference in CSP over time (Group × Time interaction effect, *F* statistic) between the exercise and control groups. The parameters of this calculation were as follows: estimated effect size = 0.25, *p* < 0.05, power = 0.80, required sample: *N* = 36 (*n* = 18 per group).

### TREATMENT GROUPS

#### Exercise group

Participants in the exercise group each received an identical 60-min low to moderate intensity exercise routine conducted at the hospital gymnasium. A certified personal trainer under the supervision of a certified exercise physiologist guided the sessions. Exercise sessions consisted of 5 min of light, callisthenic warm-up and stretching, 25 min of low-impact aerobics, 25 min of resistance training, and a 5-min cool-down including light stretches. The aerobic exercise was conducted using a low-impact, step-aerobic video to standardize the activities. Participants were evaluated during the exercise session using a heart rate monitor to maintain the prescribed aerobic intensity of 40–60% of heart rate reserve. The resistance training consisted of five exercises performed at an intensity of 8–12 repetition maximum, and included: seated row, squat, chest press, shoulder press, and abdominal crunches on a stability ball.

#### Control group

Participants in the control group were required to sit quietly in front of a computer monitor where they watched 60 min of emotionally neutral television programming, consisting of 6 min × 10 min episodes of Walt Disney’s Silly Symphony Cartoons [these programs have been previously used for inciting neutral neurological stimulus, i.e., neurological rest (Guglietti et al., 2013)]. Participants allocated to the control group were offered an exercise session similar to the exercise group following the study.

### ASSESSMENTS

Participants completed two outcome measure assessments: before randomization (T1) and immediately after their intervention or control assignment (T2). At both time points, participants completed a package of questionnaires and TMS for CSP.

### OUTCOMES

The primary outcome of this study was the duration of the CSP. TMS was applied to the hand area of the left motor cortex with a figure-of-eight magnetic coil and two Magstim 200 magnetic stimulators (Magstim, Whitland, Dyfed, Wales). The coil diameter was 70 mm for each loop. The coil was held tangentially on the head with the handle pointing backward and 45∘ laterally from the midline. Surface electromyographic recordings of the right APB were collected using dedicated software (Cambridge Electronics Design, UK), using disposable disk electrodes placed in a tendon-belly arrangement over the bulk of the muscle. Subjects were asked to maintain relaxation throughout the experiments and the EMG was monitored on a computer screen and via speakers at high gain. Each TMS session consisted of the establishment of the participant’s resting motor threshold of the right APB, followed by the CSP paradigm. TMS testing was conducted by a blinded technician, trained and experienced in TMS and CSP testing. Measurement of the contralateral CSP duration was obtained in moderately tonically active right APB (i.e., 20% of maximum contraction) by stimulating the left motor cortex with intensities of 140% of resting motor threshold. This intensity was chosen based on evidence that suggests that CSP duration at the higher stimulus intensities (140% of motor threshold) mainly reflects the activation of GABA_B_ receptor-mediated inhibitory neurotransmission (Chamberlain et al., 2006). Ten trials were performed. The CSP duration was the time from the motor evoked potential onset to the return of any voluntary EMG activity. This is referred to as the absolute CSP and ends with a deflection in the EMG waveform (Tergau et al., 1999). The CSP was determined with a previously published automated method (Daskalakis et al., 2008).

Several self-report measures of mood were implemented to further assess acute exercise-related responses. The Exercise-Induced Feeling Inventory (EIFI; Gauvin and Rejeski, 1993) is a 12-item measure that assesses acute exercise-related feelings using four subscales: positive engagement, revitalization, tranquility, and physical exhaustion. The EIFI demonstrated strong internal consistency and reliability coefficients (Gauvin and Rejeski, 1993). The Profile of Mood States-Short Form (POMS; Shacham, 1983) assesses mood based on six factor-based subscales, derived from the original scale (McNair et al., 1971): tension–anxiety, depression–dejection, anger–hostility, fatigue–inertia, vigor–activity, and confusion–bewilderment with a seventh score of Total Mood Disturbance (calculated by subtracting the score on the one positively scored subscale, vigor–activity, from the sum of the other five subscales; McNair et al., 1971). The short form version is highly correlated across all subscales with the original, long-version (Schoenfeld et al., 2013). The State-Trait Anxiety Inventory (STAI; Spielberger et al., 1983) is a brief, 40-item self-report assessment of state and trait anxiety in adults. This measure is reliable and valid, and concordance with other measures of anxiety (Peterson and Heilbronner, 1987). To assess depression and anxiety we used the Hospital Anxiety and Depression Scale (HADS; Zigmond and Snaith, 1983). Construct validity was demonstrated by the two scales correlating well with psychiatric clinical interview ratings (depression, *r* = 0.70 and anxiety, *r* = 0.74, *p* < 0.001) and by responsiveness to a counseling intervention (Johnston et al., 2000).

Demographics (e.g., age, educational status, etc.) and other information believed to have a possible influence on outcomes (tumor and treatment characteristics) were collected and compared across treatment conditions. Baseline physical activity volume was measured to assess its relationship with baseline CSP. Physical activity volume was measured using the Godin Leisure-Time Exercise Questionnaire (Godin and Shephard, 1985) which is a 3-item measure that assesses the frequency of mild, moderate and strenuous bouts of exercise performed for at least 15 min in duration during a typical week. The Godin Leisure-Time Exercise Questionnaire has been successfully used in exercise studies with PCa survivors (Culos-Reed et al., 2010), and an independent evaluation confirmed its reliability/validity compared to nine other self-report measures of exercise (Jacobs et al., 1993).

### STATISTICAL ANALYSIS

Baseline between-group comparisons were performed using independent samples *t*-test for continuous variables and chi-squared analyses for categorical variables. For the primary and secondary outcomes, ANCOVA were conducted to compare differences in CSP and questionnaire data at post-intervention between-groups (exercise versus control), controlling for the baseline value of the outcome of interest. Pearson correlation coefficients were used to assess the relationship between self-reported mood and age with CSP at baseline. Pearson correlation coefficients were also conducted for T1–T2 difference (change score) in self-reported mood and CSP for the exercise and control groups. We conducted two exploratory analyses. First, we assessed the effect of meeting the ACSM physical activity guidelines for cancer survivors (150 min of moderate intensity exercise per week) on CSP (baseline value) using independent samples *t*-test. Second, we assessed the effect of ADT on CSP (baseline value) using independent samples *t*-test. Data were analyzed using the Statistical Package for Social Sciences version 19.0 (IBM, Armonk, NY, USA).

## RESULTS

**FIGURE 1:**
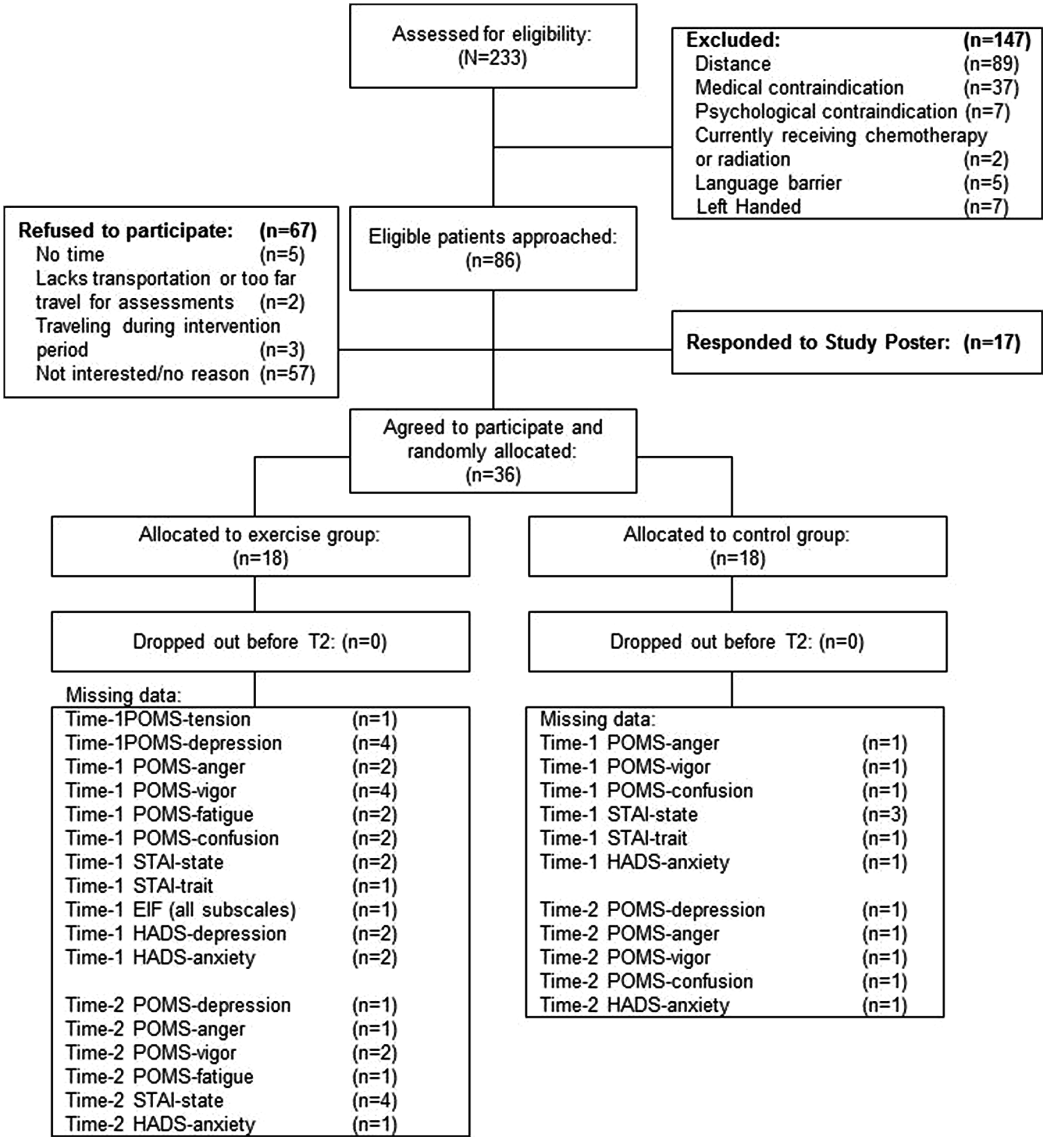
**CONSORT diagram**.

The CONSORT diagram is presented in **Figure 1**. Eighty-six eligible participants were approached for participation and 19 agreed (22% participation rate). Seventeen participants responded to the study poster. The 36 participants were randomly assigned to the exercise (*n* = 18) or control (*n* = 18) group.

Baseline participant characteristics are presented in **Table 1**. The exercise and control groups were different at baseline in age [exercise: 67.7 years (±7.6) versus control: 61.6 years (±9.1); *p* = 0.04]. The groups were similar in all demographic measures, treatment characteristics, and outcome measures (*p* > 0.05). There were no adverse health events related to the exercise or TMS procedures.

**Table 1:** Demographic characteristics of study participants.

		Exercise (*n* = 18)	Control (*n* = 18)	*p*-value
Age (years)		67.7 (7.6)	61.6 (9.1)	0.035
Physical activity volume (min/week)		362.1 (315.4)	420.6 (249.5)	0.541
Ethnicity				
	Caucasian	16 (88%)	15 (83%)	0.192
	South East Asian/East Asian	0	3 (17%)	
	African–Canadian	1 (6%)	0	
	Other	1 (6%)	0	
Marital status				
	Married	12 (67%)	13 (72%)	0.814
	Single (including widowed, separated, divorced)	6 (33%)	5 (28%)	
Annual income				
	<$40k	4 (22%)	4 (22%)	0.394
	$40–$80k	6 (33%)	3 (17%)	
	>$80k	8 (44%)	10 (61%)	
Education				
	Less than high-school	1 (5%)	0	0.275
	High-school degree	6 (33%)	2 (11%)	
	College/trade school	3 (17%)	1 (5%)	
	University degree	3 (17%)	6 (33%)	
	Graduate degree	4 (23%)	8 (46%)	
	Other	1 (5%)	1 (5%)	
Employment				
	Retired	9 (50%)	5 (28%)	0.131
	Working part time	4 (22%)	2 (11%)	
	Working full time	5 (28%)	11 (61%)	
Currently smokes tobacco		6 (33%)	4 (22%)	0.392
Primary cancer treatment				
	Radical prostatectomy	12 (67%)	14 (78%)	0.730
	External beam radiation	4 (22%)	3 (17%)	
	Androgen deprivation therapy	2 (11%)	1 (5%)	
Currently receiving androgen deprivation therapy		9 (50%)	5 (28%)	0.171

*Data on continuous variables are presented as Mean (SD); *p*-value for between-group differences of continuous variables using independent sample *t*-test. Data for categorical variables are presented as Frequency (% of group); *p*-value for χ^*2*^.*

The effect of the exercise session on primary and secondary outcomes is presented in **Table 2**. There were no significant main effects for group or time, and no significant interaction effects for any of the group comparisons. There was a trend toward reduced tension (*F*_(1,32)_ = 3.885; *p* = 0.057) and increased positive engagement (*F*_(1,32)_ = 2.967; *p* = 0.095) in the exercise group compared to the control group.

Given the observed baseline between-group differences in age, we conducted a secondary analysis with age as a covariate in the ANCOVA. There was a trend toward increased positive engagement in the exercise group compared to the control group (*F*_(1,31)_ = 2.928; *p* = 0.097). There were no significant findings for our other outcomes (*p* < 0.05).

**Table 2:** Acute effects of exercise on CSP and psychosocial outcomes.

	Δ(T2–T1)		Between-groups difference in mean change from T1 to T2	
Variable	Exercise	Control	*F*	*p*
CSP (sec)	0.001 (0.025)	0.006 (0.019)	1.222	0.277
STAI-state	0.36 (4.31)	1.07 (2.87)	0.205	0.655
STAI-trait	–0.29 (3.37)	1.06 (5.45)	0.538	0.469
POMS-tension	–2.71 (3.27)	–1.72 (2.14)	3.885	0.057
POMS-depression	–0.50 (2.84)	–0.94 (1.78)	1.569	0.375
POMS-anger	–1.47 (1.64)	–1.88 (2.45)	0.192	0.664
POMS-vigor	0.23 (3.81)	–0.59 (4.49)	0.241	0.627
POMS-fatigue	–0.84 (2.93)	–0.78 (2.41)	0.131	0.720
POMS-confusion	–1.56 (3.20)	–0.82 (1.24)	0.920	0.345
EIFI-positive engagement	1.35 (2.29)	–0.39 (3.91)	2.967	0.095
EIFI-revitalization	1.76 (4.07)	0.57 (3.36)	1.662	0.207
EIFI-tranquility	0.88 (1.90)	0.50 (3.13)	0.535	0.470
EIFI-exhaustion	0.76 (2.39)	–0.17 (2.30)	1.477	0.233
HADS-anxiety	0.44 (1.41)	0.18 (1.01)	0.105	0.748
HADS-depression	0.81 (2.73)	0.33 (1.92)	0.375	0.545

*Data are presented as mean (standard deviation); Between-groups analysis:p-value reported for ANCOVA when adjusting for baseline value. CSP, cortical silent period; EIFI, Exercise-Induced Feeling Inventory; HADS, Hospital Anxiety and Depression Scale; POMS, Profile of Mood States – Short Form; STAI, State-Trait Anxiety Inventory; T1, Time Point 1 (baseline); T2, Time Point 2 (post-test).*

A statistically significant positive correlation was observed between CSP and the trait anxiety subscale of the STAI at baseline (*r* = 0.48, *p* = 0.004; i.e., longer CSP was associated with increased anxiety). Age and CSP shared a significant, low-moderate negative correlation (*r* = –0.34, *p* = 0.041). There were no other significant correlations at baseline. In the exercise group, there was a strong, negative correlation between the change-scores of CSP and the vigor subscale of the Profile of Mood States (*r* = –0.714, *p* = 0.006). There were no other significant correlations in the exercise or control groups.

Thirty participants met the ACSM’s physical activity guidelines with no difference in the frequency of those meeting or not meeting the guidelines in the exercise and control groups (χ^2^ = 3.2, *p* = 0.074). There was no difference in baseline CSP between those meeting the guidelines and those that did not [meeting guidelines: 0.108 s (±0.028) versus not meeting guidelines: 0.107 s (±0.018); *p* = 0.975].

Fourteen participants were currently receiving ADT. Participants receiving ADT had a significantly shorter CSP than participants not receiving ADT [on-ADT: 0.961 s (±0.029) versus off-ADT: 0.115 s (±0.023); *p* = 0.035]. In bivariate correlational analyses restricted to participants that were not receiving ADT, CSP was positively correlated with the trait anxiety subscale of the STAI (*r* = 0.543, *p* = 0.011). There were no other significant (i.e., *p* < 0.05) bivariate correlations. When completing the ANCOVAs of the effect of exercise versus controls on CSP in participants who were off ADT (*n* = 22), there was no significant interaction effect of exercise on CSP (*F*_(1,19)_ = 0.157, *p* = 0.697), while the main effect of the group was related to baseline differences in CSP (*F*_(1,19)_ = 11.75, *p* = 0.003).

## DISCUSSION

To our knowledge, this is the first study to assess the acute psychological and neurophysiological effects of exercise in a population of cancer survivors. While, Fisher et al. (2008) previously demonstrated lengthened CSP with high-intensity exercise training in participants with Parkinsons’s disease, ours was the first assessment of exercise effects, CSP, and mood in a single study. Fisher et al. (2008) found functional improvements in patients with Parkinson’s disease of gait speed, step and stride length, and hip-ankle joint excursion and improved weight distribution during sit-to-stand tasks, but they did not assess mood. Our study found that one acute bout of exercise did not alter CSP in PCa survivors whereas the CSP lengthening effects demonstrated by Fisher et al. (2008) required 24 high-intensity exercise sessions over 8 weeks. Accordingly, it may be that exercise-related CSP effects, if they occur reliably, require more extensive, consistent training than a single bout. Further research is warranted regarding the CSP effects of exercise as recent animal studies suggest anxiety-reducing exercise effects linked to the same GABAergic responses monitored through CSP (Schoenfeld et al., 2013).

Novel acute relationships between CSP and psychological measures were observed, namely a positive correlation between CSP and trait anxiety. The relationship between CSP and trait anxiety may be characteristic of a group of patients with a history of PCa, in their seventh; and several were actively androgen deprived (*n* = 14), a treatment often used for patients with advanced disease. Consequently, this group may have chronic elevations in anxiety but with a longer CSP as a result of adapting to life with routine monitoring for disease progression and high risks of receiving terminal disease prognoses. The longer CSP may be associated with greater neuroinhibition, and in contrast with the greater levels of neuroexcitation, likely associated with more impulsive behavioral-response styles. Those survivors who are more reflective might be more realistic about heightened mortality risk and therefore more accustomed to living with mortality anxiety as a regular feature of current lifestyle. Conversely, CSP was not related to state anxiety, a measure of more immediate (situational) anxiety, further suggesting that these men were chronically anxious, not situationally anxious. Notwithstanding these intriguing relationships, cautious interpretation of these correlative findings is warranted given that they were secondary analyses of a relatively small sample and the number of correlational analyses increases the risk for Type 1 error.

We observed no effect of meeting the ACSM guidelines of physical activity on CSP, suggesting that CSP may not be a marker for chronic adherence to an exercise program at the level prescribed by ACSM. CSP was significantly shorter in men that were treated with ADT than those who were not, a finding that contributes to the uncertainty of findings in previous research on the role of testosterone on cortical excitability and inhibition. In a study by Bitran et al. (1993), testosterone exposure was found to be associated with increased anxiolytic behavior as well as inhibition via GABAergic pathways in rats. By extension, a *decrease* in testosterone via ADT would be expected to inhibit these same GABAergic pathways, leading to a disinhibited level of cortical excitability. Consistent with this, our findings suggest that ADT may lead to a decreased cortical inhibition as evidenced by the significantly shortened CSP in men that were treated with ADT compared to those that were not. This result contributes evidence addressing the controversial topic of whether testosterone is associated with cortical excitation (Bonifazi et al., 2004) or inhibition (Bitran et al., 1993; Herzog et al., 1998).

Finally, we found a negative correlation between changes in CSP and vigor in the exercise group only (longer CSP correlated with reduced vigor) which may indicate that the men who had exercised most vigorously for their capacities (therefore lengthening CSP) were most fatigued at the post-exercise data point (about 15 min after the exercise bout concluded). The invigorating effect that might be associated with more long-term commitment to exercise may not have appeared yet for these men.

Our study has methodological strengths. First, it was an RCT adequately powered to detect changes in CSP between the control and exercise groups. Second, our CSP assessors were blinded to group assignment. Third, we standardized exercise activity using an aerobic exercise video and resistance training routine that had strict parameters for training volume (repetitions, sets, heart rate, etc.). Fourth, we utilized both objective and subjective validated measures of psychological well-being. In particular, use of objective measures of cortical physiology believed to be significantly associated with psychological status can circumvent some response biases (placebo effect, social desirability, recall bias) confounding self-report measures.

Our study must also be considered in light of several limitations. First, we observed a between-group difference in age despite randomization; however, we adjusted for these differences in a secondary analysis that did not yield significant differences with the unadjusted model. The findings that CSP was not significantly increased with exercise could be explained by the characteristics of the aging motor cortex during physically complicated tasks, but a larger sample size of participants may be necessary in future research to detect a significant difference. Inclusion of a young adult control group may be advised. Further to the point on how aging affects CSP in a way that could potentially interact with the effects of exercise, researchers have previously shown that CSP relates with age. CSP was shown to differ in participants who had a 45 year gap (26 versus 71 years; Oliviero et al., 2006). In contrast, our participants are approximately 6 years apart in age. Further research is required on age-related changes in CSP.

A second limitation is that we did not control or measure the amount of time between the CSP measurements and the exercise or control activities. The exercise room was in an adjacent building, an approximately 5-min walk from the CSP measurement room, compared to the video-watching room which was on the same floor as the CSP measurement room. While no studies have examined any possible post-exercise declines of exercise-related effects on CSP, future studies should measure and control for the amount of time between exercise cessation and CSP measurement. However, in our study the post-test CSP was measured between 5 and 15 min after the intervention or control condition. Third, we included participants that were and were not actively undergoing ADT for PCa. While ADT was associated with a shorter CSP, we did not observe any effect of exercise-related to ADT status and the sample sizes of these sub-analyses were too small to be conclusive. Fourth, the low to moderate intensity of our exercise program may not have been sufficiently intense to stimulate significant changes in CSP. Previous measures of exercise on brain activity have demonstrated potential intensity-related differences (Nybo and Nielsen, 2001; Kamijo et al., 2004). Future studies should examine more intense training protocols for possible effects on CSP. Fifth, although we screened medical records and inquired about mental health history and treatments prior to participation, our screening of cognitive states may have been improved with the use of brief, validated measures such as the Mini-Mental State Examination (Folstein et al., 1975), and is recommended for future trials. Moreover, we excluded men who were using of anxiolytic and antidepressive medications as proxies for the presence of mood disorders, but did not confirm the absence of any psychopathology with a formal psychological assessment. Future trials would benefit from a formal evaluation of psychological states to reduce the possibility of confounding outcomes. Finally, we did not collect body composition measurements (e.g., height, weight) that at present do not appear to have a direct effect on CSP or post-exercise mood; however, they may have an indirect effect whereby the effort required to complete exercise is distinctly different in overweight or obese participants compared to healthy weight participants.

With previous studies in other populations suggesting that improvements in depression and anxiety could be attained with physical exercise and meditation, we hypothesized that one bout of acute exercise would be able to evoke similar responses in men with PCa and concomitant increases in CSP. Given that we did not find effects of acute, low to moderate exercise on CSP or anxiety and depression, it may be that longer, more frequent, or more intense sessions are required to induce changes in measures of cortical inhibition, especially those related to longer-acting mechanisms, such as CSP. More research in this field is required to clarify the role of exercise on the CSP.

## AUTHOR CONTRIBUTIONS

Daniel Santa Mina: study conception, principal investigator, primary writer; Crissa L. Guglietti: study conception, outcome assessor, statistical analyses; Danilo R. de Jesus: participant screening, safety monitoring during TMS and CSP, secondary writer; Saam Azargive: participant recruitment, intervention delivery support, secondary writer; Andrew G. Matthew: participant recruitment, final approval of manuscript, critical for intellectual content; Shabbir M. H. Alibhai: participant recruitment, final approval of manuscript, critical for intellectual content; John Trachtenberg: participant recruitment, final approval of manuscript, critical for intellectual content; Jeffrey Z. Daskalakis: study conception, critical for intellectual content, laboratory usage, final approval of manuscript; Paul Ritvo: study conception, critical for intellectual content, final approval of manuscript.

## Conflict of Interest Statement

The authors declare that the research was conducted in the absence of any commercial or financial relationships that could be construed as a potential conflict of interest. All procedures followed were in accordance with the ethical standards of the responsible committee on human experimentation (institutional and national) and with the Helsinki Declaration of 1975. Informed consent was obtained from all patients for being included in the study.

## Abbreviations

ACSM: ACSM,AmericanCollege of SportsMedicine
ADT: androgen deprivation therapy
ANCOVA: analysis of covariance
APB: abductor pollicis brevis muscle
BOLD: blood oxygen level dependent
CONSORT: consolidated standard for reporting trials
CSP: cortical silent period
GABA: gamma-aminobutyric acid
EEG: electroencephalogram
M1: primary motor cortex
mm: millimeter
ms: milliseconds
PCa: prostate cancer
RCT: randomized controlled trial
s: seconds
SMA: supplementary motor area
TMS: transcranial magnetic stimulation
